# How do people use and view infographics that summarise health and medical research? A cross-sectional survey

**DOI:** 10.1186/s12909-022-03744-6

**Published:** 2022-09-14

**Authors:** Joshua R. Zadro, Giovanni E. Ferreira, Mary O’Keeffe, Will Stahl-Timmins, Mark R. Elkins, Christopher G. Maher

**Affiliations:** 1 Institute for Musculoskeletal Health, The University of Sydney and Sydney Local Health District, PO Box M179, Level 10 North, King George V Building, Royal Prince Alfred Hospital, Missenden Road, Camperdown, NSW 2050 Australia; 2 Data Graphics Designer, The BMJ, London, UK; 3 Faculty of Medicine & Health, The University of Sydney, Sydney, Australia

**Keywords:** Infographics, Visual abstract, Graphical abstract, Health, Medicine, Cross-sectional, Survey

## Abstract

**Background:**

Understanding how people use infographics and their opinion on them has important implications for the design of infographics but has not been investigated. The aim of this study was to describe people’s use of and opinions about infographics summarising health and medical research, preferences for information to include in infographics, and barriers to reading full-text articles.

**Methods:**

We conducted an online cross-sectional survey of consumers of infographics that summarise health or medical research. Demographic and outcome data were collected and summarised using descriptive statistics. A sensitivity analysis explored whether being a researcher/academic influenced the findings.

**Results:**

Two hundred fifty-four participants completed the survey (88% completion rate). Participants included health professionals (66%), researchers (34%), academics (24%), and patients/the public (13%). Most used Twitter (67%) and smartphones (89%) to access and view infographics, and thought infographics were useful tools to communicate research (92%) and increase the attention research receives (95%). Although most participants were somewhat/extremely likely (76%) to read the full-text article after viewing an infographic, some used infographics as a substitute for the full text at least half of the time (41%), thought infographics should be detailed enough so they do not have to read the full text (55%), and viewed infographics as tools to reduce the time burden of reading the full text (64%). Researchers/academics were less likely to report behaviours/beliefs suggesting infographics can reduce the need to read the full-text article.

**Conclusions:**

Given many people use infographics as a substitute for reading the full-text article and want infographics to be detailed enough so they don’t have to read the full text, a checklist to facilitate clear, transparent, and sufficiently detailed infographics summarising some types of health and medical research may be useful.

**Supplementary Information:**

The online version contains supplementary material available at 10.1186/s12909-022-03744-6.

## Introduction

‘Infographic’ is an abbreviated term for an information graphic [1]. Infographics generally combine text, images and data visualisations to present information visually, increase the attention it receives, and to improve comprehension and recall [1–3]. Infographics are becoming increasingly popular as a method for summarising research findings [3–5], although they often have other uses in research (e.g. present information more visually or interactively, highlight certain information from a research article). Many health and medical journals now use infographics, such as visual abstracts, graphical abstracts and interactive graphics, to boost the visibility and dissemination of the research they publish [6] (e.g. New England Journal of Medicine, The BMJ, JAMA Oncology, British Journal of Sports Medicine).

Infographics appear to be increasing in popularity [1] and evidence suggests some infographics increase the attention an article receives on social media [6–8]. However, it is unknown whether people use different types of infographics more commonly as stand-alone resources to interpret research findings or to decide whether to seek more information about a study. Either approach could explain why some infographics decrease or have no effect on the attention full-text articles receive in some cases [3, 8–10]. There is evidence of harmful misuse of research [11] when clinicians only read the abstract of an article. For example, the President of Global Strategies for HIV Prevention (Arthur Amman) tells a story of a physician engaged in perinatal HIV prevention in southern Africa who started delivering a less effective preventative treatment because they only had access to the abstract of an article, which spun the study’s results. The physician’s decision to alter their prevention approach based on the article’s abstract may have harmed many people [11]. We are concerned a similar scenario could occur if health professionals, researchers, or patients view infographics as stand-alone resources. These concerns are compounded by our recent work showing that most infographics from health and medical journals do not report enough information to allow readers to adequately interpret and apply study findings (e.g. key study characteristics, limitations, effect sizes) [1]. Conversely, if research is not accessible because it takes too long to read for overwhelmingly busy clinicians or is too challenging for many patients to understand, valuable health innovations could be under-utilised.

Understanding people’s use of and opinions about infographics has important implications for their design but has not been investigated. Infographics won’t ever be able to replace the full-text article. However, if most people use infographics as stand-alone resources or substitutes for reading the full-text article, there may be a need to ensure information presented in infographics is more comprehensive (i.e. through enhanced visuals or text) and reflects important features of the full-text article. People may also have different opinions about the functions of infographics, the type of information they expect to see, and experience barriers to reading full-text articles which should be considered when designing infographics. The aims of this study were to describe how people use infographics that summarise health and medical research, their opinions about these infographics, their preferences for what information to include in these infographics, and barriers to reading full-text articles that might lead them to solely rely on an infographic.

## Methods

### Study design, participants, and recruitment

We conducted an online cross-sectional survey, with data collected between August and December 2021. We posted a link advertising our study on Twitter and Facebook to recruit people who self-identified as consumers of infographics summarising health or medical research (e.g. health professionals, researchers, patients or members of the public). People had to be 18 years or older to participate and could be living in any country. Study advertisements were posted from the Institute for Musculoskeletal Health Twitter (@msk_health; 4500 followers) and Facebook (@IMHSydney1; 150 followers) accounts, which reach a mix of researchers, academics, health professionals, and members of the public. These accounts mostly post, retweet or share the findings of interesting musculoskeletal research. They were not posting infographics produced by the Institute for Musculoskeletal Health at the time this survey was conducted, although infographics from journals are occasionally retweeted or shared.

### Data collection

The Twitter and Facebook post included a link to complete an online survey hosted in Qualtrics survey software. The first page of the survey briefly described the study, provided a link to read the Participant Information Statement (which defined infographics as per the opening sentences of this manuscript), and included a Participant Consent Form. There was no financial incentive for participants. All recruitment and data collection procedures were approved by the University of Sydney Human Research Ethics Committee (Reference number: 2021/542). All methods were carried out in accordance with relevant guidelines and regulations. Before starting the survey, all participants provided consent by checking a box that confirmed they had read the Participant Information Statement and Consent Form and agreed to participate. Participants’ rights were protected. Informed consent was obtained from each participant.

Participants were asked to provide demographic data such as age, gender, educational attainment, employment status, and background (researcher, health professional, academic, patient or member of the public, other). Participants were asked: “When did you last come across an infographic that summarised research you were interested in (e.g. on social media, in a journal)?” Response options included: “In the past week”; “In the past month”; “In the past 6 months”; “In the past 12 months”; “I have never come across an infographic that summarised research I was interested in”. Participants who selected the last option were excluded from the study. The full survey is in Additional file 1.

### Outcomes

#### Use of infographics

This included questions on how likely participants are to find and read the full text article after viewing an infographic (5-point Likert scale from ‘Extremely unlikely’ to ‘Extremely likely’), how often they use infographics as a substitute for reading the full text article (5-point Likert scale from ‘Never’ to ‘Always’), and how participants access (e.g. social media, journals) and view infographics (e.g. smart phone, laptop).

#### Opinions about infographics

Participants were asked whether they think infographics should be detailed enough so readers can translate the findings to their context without having to read the full-text article (5-point Likert scale from ‘Definitely not’ to ‘Definitely yes’), whether they think infographics are useful tools for communicating research and increasing the attention research receives (5-point Likert scale from ‘Definitely not’ to ‘Definitely yes’), and what they think the functions of an infographic should be. This last question included pre-specified options (e.g. ‘reduce the time burden of reading the full-text article’, ‘entice people to read the full-text article’) and a free-text box for participants to list other functions.

#### Preferences for information to include in infographics

Participants were asked to indicate what information from the full-text article should be included in an infographic (e.g. ‘conclusion or ‘take away’ message’, ‘description of the population, intervention(s), comparison(s) and outcome(s)', ‘sample size’).

#### Barriers to reading full-text articles

Participants were asked to indicate what barriers they experience when trying to read full-text articles.

The survey finished with a free-text response question where participants could add any other comments that would help the researchers understand how they use or view infographics (Additional file 2).

### Data analysis

All data were summarised descriptively using counts and percentages, means and standard deviations (SD), and median and interquartile ranges (IQR), as appropriate. We performed a sensitivity analysis summarising outcome data for participants involved (vs. not involved) in research and/or academia since these groups may use infographics for different reasons (e.g. due to a lack of access or time to read full-text articles). We used a Chi^2^ test to investigate differences in outcomes between these groups and conducted analyses using Stata BE Version 17.1 (StataCorp LLC, College Station, Texas, USA). Responses to the free-text question at the end of the survey were grouped into themes (Additional file 2).

## Results

### Sample characteristics

88% of those who commenced the survey completed it (*n* = 254/289). Table 1 reports the characteristics of participants who completed the survey (n = 254). These participants completed the survey in a median time of 7 minutes (IQR 5 to 10).

**Table 1:** Demographics of the participants who completed the survey (*N* = 254)

Demographics	Descriptive statistics
**Female, n (%)**	116 (46%)
**Age (years), mean (SD)**	37 (12)
**Education, n (%)**	
High school (completed)	12 (5%)
Non-university tertiary education	6 (2%)
University	236 (93%)
**Employment, n (%)**	
Employed	217 (85%)
Student	32 (13%)
Unemployed or retired	5 (2%)
**Background, n (%)**^**a**^	
Researcher	85 (33%)
Academic	62 (24%)
Health professional	174 (69%)
Patient or member of the public	25 (10%)
Researcher or academic	116 (46%)
Not involved in research or academia	138 (54%)
**Last come across an infographic, n (%)**	
In the past week	193 (76%)
In the past month	47 (19%)
In the past 6 months	13 (5%)
In the past 12 months	1 (< 1%)

*n* number of participants satisfying the item, *N* number of participants with data, *SD* standard deviation

^a^Percentages do not add to 100% because participants could select multiple options

### Use of infographics

Most participants were somewhat/extremely likely to find and read the full-text article after viewing an infographic (76%). Some used infographics as a substitute for reading the full-text article at least half of the time (41%), and this was more common among those not involved (vs. involved) in research or academia (53% vs. 29%; *p* < 0.001). Most access infographics via Twitter (67%) and view infographics on a smartphone (89%). Participants not involved (vs. involved) in research or academia were less likely to access infographics via Twitter (58% vs. 78%, *p* = 0.001), more likely to access infographics via Instagram (57% vs. 29%, *p* < 0.001), and less likely to use a laptop to view infographics (47% vs. 62%, *p* = 0.017) (Table 2).

**Table 2:** Use of and opinions about infographics, preferences for information in infographics, and barriers to reading full text articles in the total sample (*N* = 254 participants) and compared between those not involved in research or academia (*N* = 138) and those involved in researcher and/or academia (*N* = 116)

	Total sample	Not involved in research/ academia	Involved in research/ academia	Chi^**2**^, ***p***-value**
**Use of infographics (primary outcomes)**				
**Likely to read full text after viewing an infographic, n (%)**				
Extremely unlikely	6 (2%)	3 (2%)	3 (3%)	4.5, *p* = 0.474
Somewhat unlikely	25 (10%)	17 (12%)	8 (7%)	
Neither likely nor unlikely	30 (12%)	14 (10%)	16 (14%)	
Somewhat likely	139 (55%)	72 (52%)	67 (58%)	
Extremely likely	54 (21%)	32 (23%)	22 (19%)	
**Use infographics as a substitute for reading full text, n (%)**				
Never	30 (12%)	9 (7%)	21 (18%)	20.8, *p* < 0.001*
Sometimes	119 (47%)	57 (41%)	62 (54%)	
About half the time	49 (19%)	31 (23%)	18 (16%)	
Most of the time	53 (21%)	40 (29%)	13 (11%)	
Always	3 (1%)	1 (1%)	2 (2%)	
Accessing infographics, n (%)*				
Twitter	170 (67%)	80 (58%)	90 (78%)	11.0, *p* = 0.001*
Instagram	111 (44%)	78 (57%)	33 (29%)	20.2, *p* < 0.001*
Journal website	87 (34%)	44 (32%)	43 (37%)	0.8, *p* = 0.386
Facebook	77 (30%)	47 (34%)	30 (26%)	2.0, *p* = 0.157
Non-journal website	42 (17%)	21 (15%)	21 (18%)	0.4, *p* = 0.537
Other	28 (11%)	9 (7%)	19 (16%)	6.2, *p* = 0.012*
Device used to view infographics, n (%)*				
Smart phone	225 (89%)	124 (90%)	101 (87%)	0.5, *p* = 0.487
Laptop	137 (54%)	65 (47%)	72 (62%)	5.7, *p* = 0.017*
Desktop	63 (25%)	31 (23%)	32 (28%)	0.9, *p* = 0.346
iPad	38 (15%)	18 (13%)	20 (17%)	0.9, *p* = 0.350
Other	3 (1%)	1 (1%)	2 (2%)	0.5, *p* = 0.463
**Opinions about infographics**				
**Infographics should be detailed enough so readers don’t have to read the full-text, n (%)**				
Definitely not	20 (8%)	7 (5%)	13 (11%)	14.0, *p* = 0.007*
Probably not	39 (15%)	17 (12%)	22 (19%)	
Might or might not	55 (22%)	24 (17%)	31 (27%)	
Probably yes	87 (34%)	53 (38%)	34 (29%)	
Definitely yes	53 (21%)	37 (27%)	16 (14%)	
**Infographics are useful tools to communicate research, n (%)**				
Definitely not	1 (< 1%)	0 (0%)	1 (1%)	11.4, *p* = 0.022*
Probably not	3 (1%)	1 (1%)	2 (2%)	
Might or might not	16 (6%)	4 (3%)	12 (10%)	
Probably yes	49 (19%)	22 (16%)	27 (23%)	
Definitely yes	185 (73%)	111 (80%)	74 (64%)	
**Infographics increase the attention research receives, n (%)**				
Definitely not	0 (0%)	0 (0%)	0 (0%)	1.1, *p* = 0.776
Probably not	4 (2%)	2 (2%)	2 (2%)	
Might or might not	8 (3%)	3 (2%)	5 (4%)	
Probably yes	53 (21%)	28 (20%)	25 (22%)	
Definitely yes	189 (74%)	105 (76%)	84 (72%)	
Functions of an infographic, n (%)*				
Communicate research in a more user-friendly way	226 (89%)	127 (92%)	99 (85%)	2.9, *p* = 0.090
Reduce the time burden of reading the full text	162 (64%)	105 (76%)	57 (49%)	19.8, *p* < 0.001*
Help readers quickly decide whether to read the full text	161 (63%)	89 (65%)	72 (62%)	0.2, *p* = 0.690
Entice readers to read the full text	146 (58%)	72 (52%)	74 (64%)	3.5, *p* = 0.062
Other	18 (7%)	9 (7%)	9 (8%)	0.1, *p* = 0.702
**Preferences for information to include in infographics**				
Information expected to see in an infographic, n (%)*				
Conclusion or ‘Take away’ message	240 (95%)	131 (95%)	109 (94%)	0.1, *p* = 0.738
Description of intervention(s)	234 (92%)	130 (94%)	104 (90%)	1.8, *p* = 0.180
Description of outcome(s)	220 (87%)	122 (88%)	98 (85%)	0.8, *p* = 0.360
Description of population	206 (81%)	105 (76%)	101 (87%)	5.0, *p* = 0.026*
Description of comparison(s)	188 (74%)	96 (70%)	92 (79%)	3.1, *p* = 0.078
Sample size	166 (65%)	84 (61%)	82 (71%)	2.7, *p* = 0.101
Statistics summarising the effect size	147 (58%)	79 (57%)	68 (59%)	0.0, *p* = 0.825
Some study limitations	100 (39%)	56 (41%)	44 (38%)	0.2, *p* = 0.667
Conflicts of interest	65 (26%)	31 (23%)	34 (29%)	1.6, *p* = 0.213
Other	14 (6%)	9 (7%)	5 (4%)	0.6, *p* = 0.442
Barriers to reading full-text articles, n (%)*				
Lack of time	196 (77%)	102 (74%)	94 (81%)	1.8, *p* = 0.178
Lack of access	180 (71%)	115 (83%)	65 (56%)	22.7, *p* < 0.001*
Unsure how to determine study quality	67 (26%)	48 (35%)	19 (16%)	11.0, *p* = 0.001*
Unsure how to interpret results	60 (24%)	37 (27%)	23 (20%)	1.7, *p* = 0.192
Unsure how to interpret methods	59 (23%)	35 (25%)	24 (21%)	0.8, *p* = 0.380
Other	14 (6%)	9 (7%)	5 (4%)	0.6, *p* = 0.442
No barriers experienced	6 (2%)	3 (2%)	3 (3%)	0.0, *p* = 0.829
Never attempted to access full text	3 (1%)	2 (2%)	1 (1%)	0.2, *p* = 0.666

NB: the ranking question was not included in the Table because the results were almost identical to the question about what information people expect to see in an infographic

*IQR* Interquartile range, *n* number of participants satisfying the item, *N* number of participants with data, *SD* standard deviation

*Percentages do not add to 100% because participants could select multiple options

**Comparison between those not involved in research or academia (*N* = 138) and those involved in researcher and/or academia (*N* = 116)

### Opinions about infographics

Many participants thought infographics should probably/definitely be detailed enough so readers can translate the findings to their context without having to read the full-text article (55%), and this was more common among those not involved (vs. involved) in research or academia (65% vs. 43%; *p* = 0.007). Most thought infographics probably/definitely were useful tools for communicating research (92%), and this was more common among those not involved (vs. involved) in research or academia (96% vs. 87%, *p* = 0.022). Most thought infographics probably/definitely were useful tools for increasing the attention research receives (95%) and viewed infographics as a way to communicate research in a more user-friendly way (89%). Most viewed infographics as a way to reduce the time burden of reading the full-text article (64%), and this was more common among those not involved (vs. involved) in research or academia (76% vs. 49%, *p* < 0.001) (Table 2). Other functions of infographics included delivering evidence-based, accessible information to patients and the public (including those with low health literacy), reaching new audiences, and using infographics as a recap after reading the full-text article.

### Preferences for information to include in infographics

Most participants expected to see a conclusion or ‘take away’ message in an infographic (95%), a description of the four PICO elements – population (81%), intervention(s) (92%), outcome(s) (87%) and comparison(s) (74%) – the sample size (65%), and statistics summarising the effects of an intervention (58%). Fewer participants expected to see study limitations (39%) and conflicts of interest (26%). Participants not involved (vs. involved) in research or academia were less likely to want a description of the population in an infographic (76% vs .87%, p = 0.026) (Table 2).

### Barriers to reading full text articles

The most common barriers to reading full-text articles were lack of time (77%) and access (71%). Less frequently reported barriers included being unsure how to determine study quality (26%), interpret the methods (23%), and interpret the results (24%). Participants not involved (vs. involved) in research or academia were more likely to report lack of access (83% vs. 56%, *p* < 0.001) and being unsure how to determine study quality as barriers (35% vs. 16%, *p* = 0.001) (Table 2).

### Other comments about use or views on infographics

The most common themes from free-text responses about use of or views about infographics were that they help communicate research in user-friendly way, inform people and support decision making, and are not all equal in quality (Additional file 2).

## Discussion

### Summary of main findings

Although most participants were somewhat/extremely likely to read the full-text article after viewing an infographic, some used infographics as a substitute for reading the full-text article at least half of the time, thought infographics should be detailed enough so they don’t have to read the full-text article, and viewed infographics as tools to reduce the time burden of reading the full-text article. Most of these behaviours and beliefs were more common among those who were not involved in research or academia. Most participants used Twitter and smartphones to access and view infographics, and thought infographics were useful tools to communicate research and increase the attention research receives. A large majority expected to see the conclusion or ‘take away’ message and the four PICO elements in infographics. Lack of time was the most common barrier to reading full-text articles in the total sample, while lack of access was the most common barrier for those not involved in research or academia.

### Strengths and weaknesses of the study

We recruited participants from a range of backgrounds (e.g. researchers, health professionals, patients, members of the public) and had a high completion rate (88%). The main limitation of this study is that data on people’s use of infographics is self-reported and may be different in real life. Another limitation is only recruiting through Twitter and Facebook, which may have led to a biased sample. For example, the high proportion of health professionals, academics, and researchers likely reflects the composition of people who follow the Institute for Musculoskeletal Health Twitter and Facebook accounts, and the type of people who were liking, re-tweeting, or sharing our study advertisement. Future research should consider ways to reach a broader audience in the medical and research community.

### Meaning of the study

Participants who were not involved in research or academia were more likely to use infographics as a substitute for reading the full-text article, think infographics should be detailed enough so they don’t have to read the full-text article, and view infographics as tools to reduce the time burden of reading the full-text article. Given that most infographics don’t allow readers to adequately interpret and apply study findings [1], these findings confirm our initial concern that some health professionals may be making poor preventative or treatment decisions because they use infographics as a substitute for reading the full-text article [11]. Nearly one-third of researchers and academics use infographics as a substitute for reading the full-text article at least half of the time, which might be due to a lack of time (reported by 4 in 5 researchers and academics). However, there could be other explanations. Some researchers and academics may use infographics to quickly scan for studies that align with their research interests or for studies that have features indicating low risk of bias (e.g. randomisation, low loss to follow-up).

Participants who were not involved in research or academia were more likely to report lack of access as a barrier to reading the full-text article, which may explain why they appear more reliant on the content of infographics. However, they were not less likely to read the full-text article after viewing an infographic compared to researchers and academics. One explanation is that participants may have interpreted our question about their likelihood of reading a full-text article after viewing an infographic in the context of having access to the full-text article. In addition, the question about barriers was framed in a way that anyone who has been unable to access a full-text article on at least one occasion would have reported ‘lack of access’ as a barrier. This may have inflated the prevalence of this barrier in our sample, particularly among those not involved in research or academia.

Another interesting finding is that participants not involved in research or academia were more likely to access infographics via Instagram (nearly twice as much) and less likely to access them via Twitter. This should be considered by journal editors and researchers trying to increase the reach of their research, particularly among non-academic audiences. In addition, a similar proportion of people involved vs. not involved in research or academia access infographics on journal websites (37% vs. 32%) which might be explained by some publishers making their infographics open access even when the full-text article is behind a paywall [12].

### Comparison to existing literature

Our participants’ view that infographics can be useful tools to communicate research is consistent with previous research highlighting that visual abstracts (a type of infographic) require less cognitive load and are more preferred over traditional abstract summarises [13] and are rated as more user-friendly [14] (without negatively impacting knowledge [14] or information retention [13]). However, it needs to be acknowledged that considerable care is required when examining research on the benefits and limitations of infographics since there is substantial variation in the design of infographics and the quality and impact of the research they summarise.

Participants’ view that infographics increase the attention research receives is also consistent with several studies that have found some infographics increased the number of impressions and re-tweets Twitter posts receive [6, 8] and increased abstract views [3, 10]. However, there are some infographics that have reduced impressions, re-tweets, abstract views and citations [9, 15]. There are also examples of some infographics that may not be superior to other research communication tools. For example, a survey of 58 physicians and nurses who were shown a one-page text summary and an infographic of a systematic review on pain medication for acute migraine found the infographic had substantially greater visual appeal, but lower clarity and comprehensibility [2].

Many participants in our study reported regularly using infographics as a substitute for reading the full-text article, thought infographics should be detailed enough so readers can translate the findings to their context without having to read the full-text article, and viewed infographics as tools to reduce the time burden of reading the full-text article. This appears to be consistent with previous research showing that some infographics either have no effect or a negative effect on article visits and full-text downloads [3, 8–10]. However, in some cases a lack of effect may be explained by low article views overall. For example, a prospective, case-control crossover study took 40 articles published over a 11-month period in *American Journal of Nephrology* and tweeted each article in three formats (citation only vs. citation plus key figure from the article vs. citation plus visual abstract) [8]. Tweets including visual abstracts received similar article visits compared to the other formats (9.0 for visual abstracts vs. 8.1 for citation only vs. 6.7 for citation plus key figure). Another study took 12 articles published in the *Canadian Journal of Emergency Medicine* that were promoted on Facebook and Twitter without infographics over a 3-month period in 2015/16 and compared these to 11 articles published in this journal and additionally promoted using infographics over the same time [3]. Articles promoted using infographics received slightly less full-text views compared to articles promoted without infographics (65 vs. 73).

In *The BMJ* between June and December 2018, tweets including visual abstracts received fewer URL clicks (29 vs. 60) compared to the average of all tweets from *The BMJ*. However, this finding was only specific to visual abstracts. Other types of infographics performed better (e.g. more detailed full page visual summaries, interactive graphics) [15]. Over the past 3 years, *The BMJ* has found that 64.9% of their tweets including visual abstracts (87.5% in 2021) have been at least 15% above the average tweet in most metrics (unpublished data). This suggests improving the quality of visual abstracts may improve their impact. Other studies have also found a positive effect of infographics on article visits [6]. Nevertheless, the total available evidence may challenge the assumption that infographics increase the attention research receives and may suggest many people are solely relying on the infographic summary of some studies to inform their research, clinical practice or healthcare choices.

### Unanswered questions and future research

There are limited data on people’s preference for what information to include in infographics and why people have certain preferences. When *The BMJ* were developing their template for visual abstracts, they sought feedback on a visual abstract of tai chi for fibromyalgia from 77 health professionals who treat patients with fibromyalgia (e.g. primary care physicians) [15]. Participants wanted more detailed statistics so they could quickly judge how effective tai chi was and for the conclusion to be at the top of the visual abstract. Possible explanations for this feedback include that these health professionals wanted infographics to reduce the time burden of reading the full-text article or that more detailed statistics may have helped them decide whether they need to read the full-text article. Our study adds further data to what people want to see in infographics, but it does not explain why people have certain preferences. Future qualitative research could be useful to explore this issue.

Given that most people sometimes use infographics as a substitute for reading the full-text article and that most broadly agree that infographics should be detailed enough so they do not have to read the full-text article, a checklist or ‘minimum standard’ for certain types of infographics may facilitate clear, transparent, and sufficiently detailed infographics summarising health and medical research. We are currently conducting a Delphi study to develop a checklist to improve the reporting of infographics summarising the main findings of comparative studies of health and medical research (e.g. randomised controlled trials, systematic reviews) [16]. Such a checklist will likely outline the minimum items necessary to include in these types of infographics (e.g. PICO items, effect size, 95% CIs) but give designers flexibility on how to present these items visually or through text. A checklist could improve the accuracy with which research findings are communicated and avoid research findings being mis-interpreted if consumers (e.g. health professionals, researchers) do not refer to the full-text article. Once developed, we will evaluate the acceptability of the checklist and whether infographics developed according to the checklist are more acceptable and lead to wiser healthcare choices. These findings will determine whether checklists for other types of infographics could be useful, but we acknowledge that checklists may be inappropriate for some types of infographics (e.g. an infographic focusing on the interaction of different participant characteristics on a secondary outcome).

## Conclusion

Although most participants were somewhat/extremely likely (76%) to read the full-text article after viewing an infographic, some used infographics as a substitute for reading the full-text article at least half of the time (41%), thought infographics should be detailed enough so they don’t have to read the full-text article (55%), and viewed infographics as tools to reduce the time burden of reading the full-text article (64%). These behaviours and beliefs were more common among those who were not involved in research or academia. A checklist to facilitate clear, transparent, and sufficiently detailed infographics summarising some types of health and medical research could improve the accuracy with which research findings are communicated and avoid research findings being mis-interpreted if readers do not refer to the full-text article.

## Supplementary Information

**Additional file 1.** Survey.

**Additional file 2.** Other comments from participants related to how they use or view infographics.

## Data Availability

All data relevant to the study are available upon reasonable request to the corresponding author.
