# Supplementary material for: How do people use and view infographics that summarise health and medical research? A cross-sectional survey
Source: BMC Med Educ. 2022 Sep 14;22:677. doi: 10.1186/s12909-022-03744-6 (PMC9472431; doi:10.1186/s12909-022-03744-6)
Supplement: Supplementary file 1 — Additional file 1. Survey. [file 12909_2022_3744_MOESM1_ESM.docx]

**Additional file 1. Survey**

**Welcome to the survey!**

This survey should not take more than 10 minutes.

**First, some questions about you…**

1. Please indicate your gender:

- Female
- Male
- Prefer not to say

1. Please indicate your age: [free text response]

___________________

1. What option best describes your highest level of education?

- Primary school completed or less
- High school (not completed)
- High school (completed)
- TAFE/Trade (completed)
- University- undergraduate degree/s (completed)
- University- postgraduate degree/s e.g. Masters, PhD (completed)
- Other (please specify) ____________________________

1. What is your employment status?

- Employed part-time
- Employed full-time
- Casual work
- Retired
- Unemployed
- Student
- Sick/disability leave
- Other (please specify) ____________________________

1. What is your background? Please select all that apply

- Researcher (please specify the field) ______________
- Academic (please specify the field) ______________
- Health professional (please specify the profession)________________
- Patient or member of the public
- Other (please specify) ____________________________

**Now some questions about infographics…**

1. When did you last come across an infographic that summarised research you were interested in (e.g. on social media, in a journal)?
   - In the past week
   - In the past month
   - In the past 6 months
   - In the past 12 months
   - I have never come across an infographic that summarised research I was interested in **[survey terminated]**
2. When you come across an infographic that summarises research you are interested in (e.g. on social media, in a journal), how likely are you to find and read the full text article?
   - Extremely unlikely
   - Somewhat unlikely
   - Neither likely nor unlikely
   - Somewhat likely
   - Extremely likely
3. How often do you use infographics summarising research as a substitute for reading the full text article?
   - Never
   - Sometimes
   - About half of the time
   - Most of the time
   - Always
4. Where do you usually find infographics? Please select all that apply
   - Facebook
   - Twitter
   - Instagram
   - Journal website
   - Non-journal website
   - Other (Please specify_______________________________)
5. What device do you usually use to view infographics? Please select all that apply
   - Smart phone
   - Laptop
   - Desktop computer
   - iPad
   - Other (Please specify_______________________________)
6. Do you think infographics summarising research should be detailed enough so readers can translate the findings to their context without having to read the full text article? (Please provide a reason for your response)
   - Definitely not (Please provide a reason for your response _______________________________)
   - Probably not (Please provide a reason for your response _______________________________)
   - Might or might not (Please provide a reason for your response _______________________________)
   - Probably yes (Please provide a reason for your response _______________________________)
   - Definitely yes (Please provide a reason for your response _______________________________)
7. Do you think infographics are useful tools for communicating research findings?
   - Definitely not
   - Probably not
   - Might or might not
   - Probably yes
   - Definitely yes
8. Do you think infographics are useful tools for increasing the attention research receives?
   - Definitely not
   - Probably not
   - Might or might not
   - Probably yes
   - Definitely yes
9. What are the functions of an infographic? Please select all that apply
   - Reduce the time burden of reading the full text article
   - Entice readers to read the full text article
   - Help readers quickly decide whether they want to read the full text article
   - Communicate research findings in a more user-friendly way
   - Other (Please specify_______________________________)
10. What information from the full text article would you expect to see in an infographic? Please select all that apply
    - A conclusion or ‘Take away’ message
    - Description of the population/participants (e.g. older adults with low back pain)
    - Description of the intervention (e.g. resistance exercise)
    - Description of the control/comparison (e.g. no treatment)
    - Description of the outcome(s) (e.g. pain, disability)
    - Sample size
    - Statistics summarising the effects of the intervention (e.g. mean differences)
    - Some limitations of the study (e.g. small sample size, lack of blinding)
    - Conflicts of interest of the study authors
    - Other (Please specify_______________________________)
11. Please rank the same infographic items in order of importance, with 1 being the most important item to include in an infographic
    1. A conclusion or ‘Take away’ message
    2. Description of the population/participants (e.g. older adults with low back pain)
    3. Description of the intervention (e.g. resistance exercise)
    4. Description of the control/comparison (e.g. no treatment)
    5. Description of the outcome(s) (e.g. pain, disability)
    6. Sample size
    7. Statistics summarising the effects of the intervention (e.g. mean differences)
    8. Some limitations of the study (e.g. small sample size, lack of blinding)
    9. Conflicts of interest of the study authors
    10. Other (Please specify_______________________________)
12. Which of the following barriers have you experienced when trying to read a full text research article? Please select all that apply
    - Lack of time
    - Lack of access to the full text (e.g. full text behind paywall)
    - Unsure how to interpret the methods
    - Unsure how to interpret the results
    - Unsure how to determine whether a paper is good or bad quality
    - Other (Please specify_______________________________)
    - I have not experienced any barriers
    - I have never attempted to access a full text research article
13. Do you have any other comments that would help us understand how you use or view infographics? __________________________________________________________
