# Supplementary material for: How do people use and view infographics that summarise health and medical research? A cross-sectional survey
Source: BMC Med Educ. 2022 Sep 14;22:677. doi: 10.1186/s12909-022-03744-6 (PMC9472431; doi:10.1186/s12909-022-03744-6)
Supplement: Supplementary file 2 — Additional file 2. Other comments from participants related to how they use or view infographics. [file 12909_2022_3744_MOESM2_ESM.docx]

| **Additional file 2. Other comments from participants related to how they use or view infographics** |
| --- |
| **Infographics help communicate research in user-friendly way** |
| Researchers don't always know how to present & "market" their findings. Infographics can really help communicate findings in user-friendly way roundly way. It is important to create amazing content that readers will remember -display data in useful engaging way. Aim to keep it simple & uncluttered with key points |
| They make research more accessible to people who are intimidated by scientific language, put the conclusions in plain language, broaden the audience. They help the impact factor too I would imagine. They are an alternative form of knowledge transfer in addition journals and conference presentations |
| I read full texts related to my research and clinical field, but not every study out there. Infographics let me know in a friendly way, what a study says about a topic I don’t usually read. |
| Increase readership reach, interest in the research |
| They are great to get a snapshot of research that is not in my subject area ie what's happening with covid. I don't have the expertise to critique an ID epidemiology paper but when a trusted person posts an infographic, I can quickly gain a high-level view of a topic |
| a good concise summary communicated important information quickly, which helps me discern which full articles to follow up on and helps me keep updated on current thought in research, without having to read every article fully |
| User-friendliness should not mean diluted, biased, or misinformed. It should be used as a means to lower the hurdles to interpreting research without misinforming |
| Infographics are easy to share with staff and create interest for further reading |
| Attention span is limited for the social media generation |
| **Infographics inform people and support decision making** |
| I think they can also be an effective way to present evidence to patients to help them make decisions |
| Infographics can be great for teaching and consumer explanations |
| Can be a great tool to guide clinicians on how to educate. Providing a streamline way of thinking and being able to convey results of research to patients in condensed and simplified way |
| Knowledge transfer to patients, students |
| They are fun to look at and can be good to print off and use to help inform people |
| Communicating policy-relevant findings to policy makers (after curating the substance behind the message) |
| really good to improve health literacy |
| Infographics can be a very useful tool for patient education |
| I use infographics as an educational tool to summarize things in a comprehensive language for the patient, allowing him to get a grasp of info and read it as many times as needed while also allowing to generate questions that I can address |
| **Not all infographics are equal in quality** |
| As with many things, the question is more nuanced. Infographics can be poorly done, or well done. That makes a huge difference. I tried to answer most questions thinking about good infographics, but really, one should be investigating what about infographics helps and what hinders understanding |
| Many a very poor quality |
| I don't read ones that are basically all text, but presented in a 'graphic' format |
| I also look for patient friendly infographics that can be used for education- not easy to find |
| They must be entertaining. Infographics tell a story. They take the place of someone telling you that story. regardless of content, they MUST entertain in some way, and possibly allow the reader to form their own opinions around the topic where appropriate |
| I think the key is to keep it simple, too much information, too many fonts, colours , arrows I find off putting |
| We need to be very careful that infographics do not propagate misinformation due to a lack of detail or selective reporting. Perhaps they should peer reviewed in conjunction with original manuscript |
| **Comments about graphics/visuals** |
| Symbols and graphics design is very important in info graphic |
| The info must provide the graphic context and meaning |
| Infographics with a black background are great for viewing at night, hopefully lessening the negative effect that white-background infographics have on sleep quality |
| See if the graphics make sense or not first. If it does, I will spend more time to read details |
| They are probably most useful as a visual representation of an abstract |
| **Different infographics are needed for different populations** |
| Different layers (more or less in-depth info), mouse overs or such might be helpful to take account of different audiences/needs. Need to take account of accessibility for people with disabilities too |
| I like using and consuming infographics about topics that are relevant to me, but that I maybe do not have much experience with |
| Infographics content depends on the call to action of the infographic, the target of the graphic, and the strength of the findings |
| I make and use infographics that summarise small parts of studies - just describing the intervention, population, or outcomes, for example. Not all infographics are designed to summarise entire studies |
| Sometimes I think it would be good to have a 'clinician version' and a 'patient version' for the same research |
| **Infographics should not be used as a substitute for reading full-text articles** |
| Use can vary quite a lot if it's an infographic of a qualitative study, infographics don't substitute reading the full-text when results are critical (eg. life or death, high cost, suggest changing longstanding practices), some worry about the many potential misuses, asking too much from an infographic makes it unreadable/too much info |
| Would be so nice if they could be a replacement but I don’t believe they can |
| Small file sizes are important for Twitter, link to full text helpful |
| **Only high-quality research should be summarised in infographics** |
| I strongly believe that rigorously conducted research should be selected as the stimulus material for infographics. If this is done there will be few limitations and conflicts of interest. The "statistics" need to be estimates not the output of statistical tests |
| When from a trusted source that I believe has critically examined full text and assessed for limitations, I can feel confident in not reading full text but must always consider the source |
| I tend to consider the source of the infographic. Typically, the ones I view come from academics with far better skills at interpreting research than I have. Almost like a trusted brand |
| **Researchers don’t have the skills to design high-quality infographics** |
| I think authors are not trained to build infographics and sometimes the result can be not informative. They should be prepared by editing professionals with authors supervision |
| Researchers are poor in both the design and use of infographics. Most as too wordy with small text and poor design. A standardized format may help but journals have no value in producing such a product |
| **Challenging to balance the amount of information with visual appeal** |
| It's a hard balance to strike to provide enough info for context and nuance without having something so cluttered it acts as a barrier to interaction |
| **Comment about where they are viewed** |
| I unusually see them via social media, and they are often presented with views of those presenting the research |
| **Infographics are used as a substitute for reading full-text articles** |
| I usually view them as a substitute to reading a full text due to lack of time. Also find it useful for patient education |
| **Infographics aren’t useful** |
| As a researcher I don't find them useful |
| **Irrelevant responses** |
| No |
| As tools to provide to patients that help support what you're saying in a trusted way |
| no |
| Many thanks for reaching out. Really appreciate the research you are doing. Happy to contribute. Keen to know the results! |
| Worthy area for further research! |
| No |
| No :) |
| no |
| Na |
